# Research on the operational properties of the soft gripper pads

**DOI:** 10.1038/s41598-024-83956-6

**Published:** 2024-12-30

**Authors:** Marcin Białek, Dominik Rybarczyk

**Affiliations:** Division of Mechatronic Devices, Institute of Mechanical Technology, Poznan University of Technology, 60-965 Poznan, Poland

**Keywords:** Grippers, Robotics, Soft Robotics, Magnetorheology, Magnetorheological Fluids, Mechanical engineering, Scientific data

## Abstract

Grippers are commonly used as a technological tooling for manipulators. They enable robots to interact with objects in their work area. Grippers have a wide range of differentiation focused on the operation performed and the properties (e.g. shape) of the object being gripped. Their design and functionality are constantly being modified, tuned and developed by both academic and industrial units. Consequently, this paper presents a proposal for a lightweight jaw using MR fluid, which can be implemented in a jaw gripper (e.g. Robotiq 2F-140) to form a hybrid soft-rigid structure. In addition, methods are presented for studying the use of soft structures in a jaw gripper. As part of the work carried out, a model of the cushion and jaw of the gripper was developed, the FEM was used to obtain the character of the deformation when the object is axially plunged into it. Experimental plunging tests as well as dynamic tests of object transfer were also carried out. The work carried out allowed to demonstrate several key aspects of the grippers area. The soft structures of the grippers should be studied in terms of the force required to deform them. This determines their applicability to fragile and deformable objects. Dynamic measurements of the handling of objects of different shapes, with simultaneous measurement of force, allow the effectiveness of the use of soft structures in the gripper to be determined. Such experiments will make it possible to determine the measurable stability and repeatability of the grasp. The results of the research and experiments will be particularly applicable to robotic arms with relatively low lifting capacity.

## Introduction

The hand, and in particular the palm, is a key element for the manipulative abilities of the human body. The versatility of its structure, and in particular the large number of degrees of freedom equal to 27^1^ , makes it possible to grasp and manipulate objects in space. Devices designed to mimic the action of the hand are called grippers. Article^2^ distinguishes between two main categories: soft and rigid, as well as four main characteristics: force exertion, precision of manipulation, number of degrees of freedom possessed and structural compliance. The article^3^ presents an overview of soft grippers. It proposes a division based on gripping methods and their effectiveness for different types of objects, defined by a scale of difficult-easy to grasp. A distinction was made between grasping as a result of: external force interaction (group one), stiffness control (group two) and adhesion control (group three). Among the first group, it can distinguish between hybrid soft-rigid comb structures^4^ ,tentacles with a comb structure ending with a suction pad^5^, and tentacles with internal air-filled chambers that determine its degree of deflection^6^. Another hybrid structure is a tentacle with a hairy surface to increase adhesion and grip^7^. It is also common to use 3D printing to build thermoplastic polyurethane (TPU) gripper components in parts^8^ or as a whole, with a harmonic^9,10^ or comb structure^11^. The second group is grippers based on stiffness control during gripping. In the paper^12^, one can find basic configurations of jamming grippers that use granules, which are often fine-grained coffee. When an object is grasped and the grains inside the balloon are adjusted to their shape, the air inside the balloon is sucked out. As a result, the shape of the grasped object is fixed. Depending on the shape and dimensions of the object, three types of grasping can be achieved. The first is based on static friction of the surfaces in contact (object-balloon). The second is accomplished by conforming to the shape of the grasped object to prevent its movement. The third is based on suction of the gripped object as a result of vacuum. These types of grip can be combined with each other. Studies include the use of different balloon and grain dimensions^13^. This includes the amount of grains that fill the balloon^14^. Importantly, in the case of grippers, the binding criterion is the pulling force or, often referred to in grippers, the holding force^15^. This is a parameter comparable to the gripping capacity of these devices. Among cushion grippers, various designs can be found in the literature that replace granules with water using a permanent magnet^16^ or a tendon^17^. The third group is grippers based on controlling adhesion and thus grip friction itself. They find their application for convex, flat and deformable objects. These include structures that resemble gecko legs^18,19^ using electroadhesion^20^, or solutions based on suction cups^21^. Other solutions can be found in the literature through structures called origami^22^. The multitude of design and modifications to existing ones are constantly expanding the possibilities of building manipulation devices^23^.

An example representing a soft-rigid hybrid is a vice gripper equipped with air pockets located on the inner surface of the jaws^24^. Depending on the shape and dimensions of the object to be grasped, it is possible to simultaneously provide all three gripping methods discussed earlier. Another example is a gripper with jaws with a three-dimensional structure on its surface^25^. This approach aims to increase adhesion compared to flat pads and is a kind of prototype of previously discussed research on the use of hairs in the tentacle gripper^7^. In this case, the aspect of external force interaction and increased adhesion can be observed. Other examples of hybrid structures are solutions based on adaptive deformable jaws^26^ or the use of anisotropic overlays on surgical needle holders designed to grasp sensitive tissues^27^. Among hybrid devices, there are solutions based on jaws equipped with adaptive cushions. The article^28^ presents gel-filled jaw pads with a silicone hemisphere inside. This solution is particularly suitable for gripping very fragile and delicate objects. By recording the pressure of the jaws, the authors were able to detect the breakage of the object and, in further research, avoid it when lifting them. The article^29^ presented a similar solution with oil-filled pads. The purpose of the research was to develop a strategy to monitor the pressure of the liquid, enabling the gripping of fragile and delicate objects without damaging them. The paper^30^ describes soft cushions that are equipped with a capacitive grid that allows measuring the deformation (deformation) of the hemispherical structure.

**Fig. 1:**
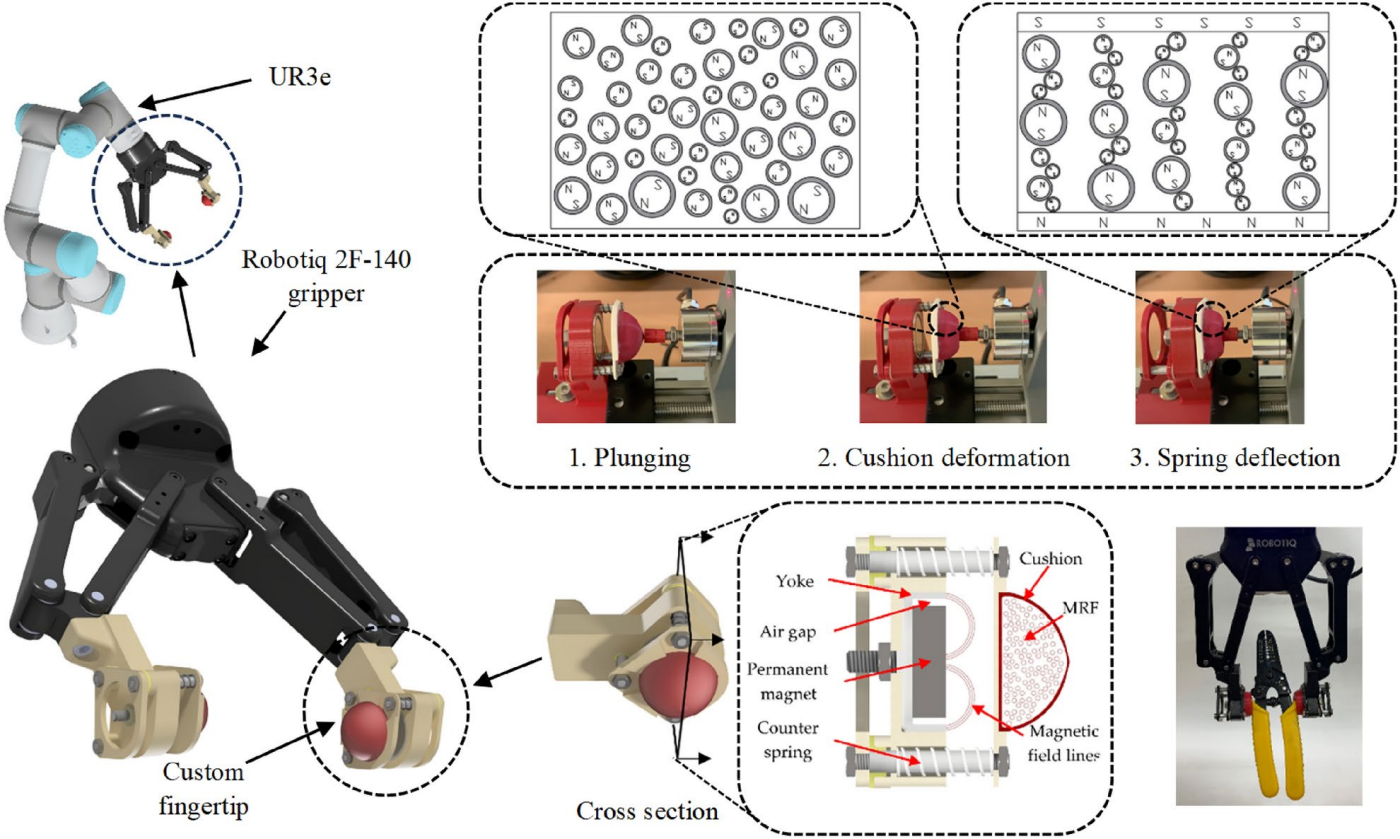
Object of the study - the jaw of the hybrid soft-rigid robotic gripper, with the cross-section indicating MR fluid, principle of operation and an example gripping application.

In the introduction of the article^31^, a classification of various grippers solutions using MR fluid was presented. Evaluation criteria were developed based on^2^. Due to the different forms of use of MR fluid in grippers, a proprietary form of classification in terms of design. Three groups were defined, taking into account the versatility of the gripper and the ability to change the orientation of the object being held. These include jaw grippers, grippers based on a “tentacle” structure, grippers based on a jamming gripper solution and those using enhanced adhesion. Among jaw grippers, a division can be seen in terms of participation of the degree of MR fluid in the gripping process. The solutions described in^32–37^ have elements that use MR fluid (such as couplings or pistons) embedded in their design. The second group, whose gripping process is fully dependent on the use of MR fluid, is based on the cushion gripper design^38–42^. Their gripping properties are fully dependent on the phenomena occurring in increasing the stiffness of the MR fluid through the insertion of a magnetic field. Hybrid solutions, on the other hand, are simultaneously present in at least two groups^42–44^. The solution described in^42^ uses the MR fluid indirectly through valves, located outside the gripper tentacles. In articles^43^ and^44^, the jaw design allows the simultaneous action of pressure (actuation) on the object, and the soft cushions limit the motion of the grasped object by changing its stiffness. This is the research area of this article. On the basis of the literature review discussed above, it can be concluded that so far:

- The force required to deform the soft structure of the gripper that is in contact with the object being gripped has not been tested. This is particularly important when gripping fragile objects;
- No method has been proposed to record the jaw contact force during the handling, on the side of the object. This approach is an alternative to solutions using tactile sensors, which register force on the side of the gripper elements;
- The aforementioned comparative study of soft-rigid hybrid structures with rigid equivalents, i.e. flat conventional jaws, used, for example, in the factory design of Robotiq’s 2F series grippers, has not been carried out;

Therefore, it was reasonable to carry out research in these scopes, both for research purposes themselves and to develop practical solutions that can be applied in industry.

The object of the study is shown in Figure 1. The solution is based on the use of magnetic field attraction and spring reaction. A detailed description is provided in^31^. The springs are at rest, providing an air gap between the magnetic field source and the cushion. As a result of the movement of the jaw, the grasped object (in this case, the pin) is plunged. Deformation occurs in the fluid cushion. Further movement of the jaw toward the object causes not only deformation of the cushion but deflection of the springs. This has the effect of reducing the air gap between the permanent magnet and the cushion. As a result, the magnetic field of the permanent magnet is inserted into the volume of the cushion and the MR fluid. The main advantages of this solution are simplicity of operation, low energy cost, lack of a coil, stability of operating temperature conditions, and, in particular, lack of additional control of the magnetic circuit. At the same time, the main objective of the application of stiffening of the cushion structure after grasping an object is preserved. Release of the grip is done in reverse. In addition, ferromagnetic particles floating in the MR fluid and the lines of force of the magnetic field generated by the permanent magnet are visualized. The field is closed by using an outer yoke.

**Fig. 2:**
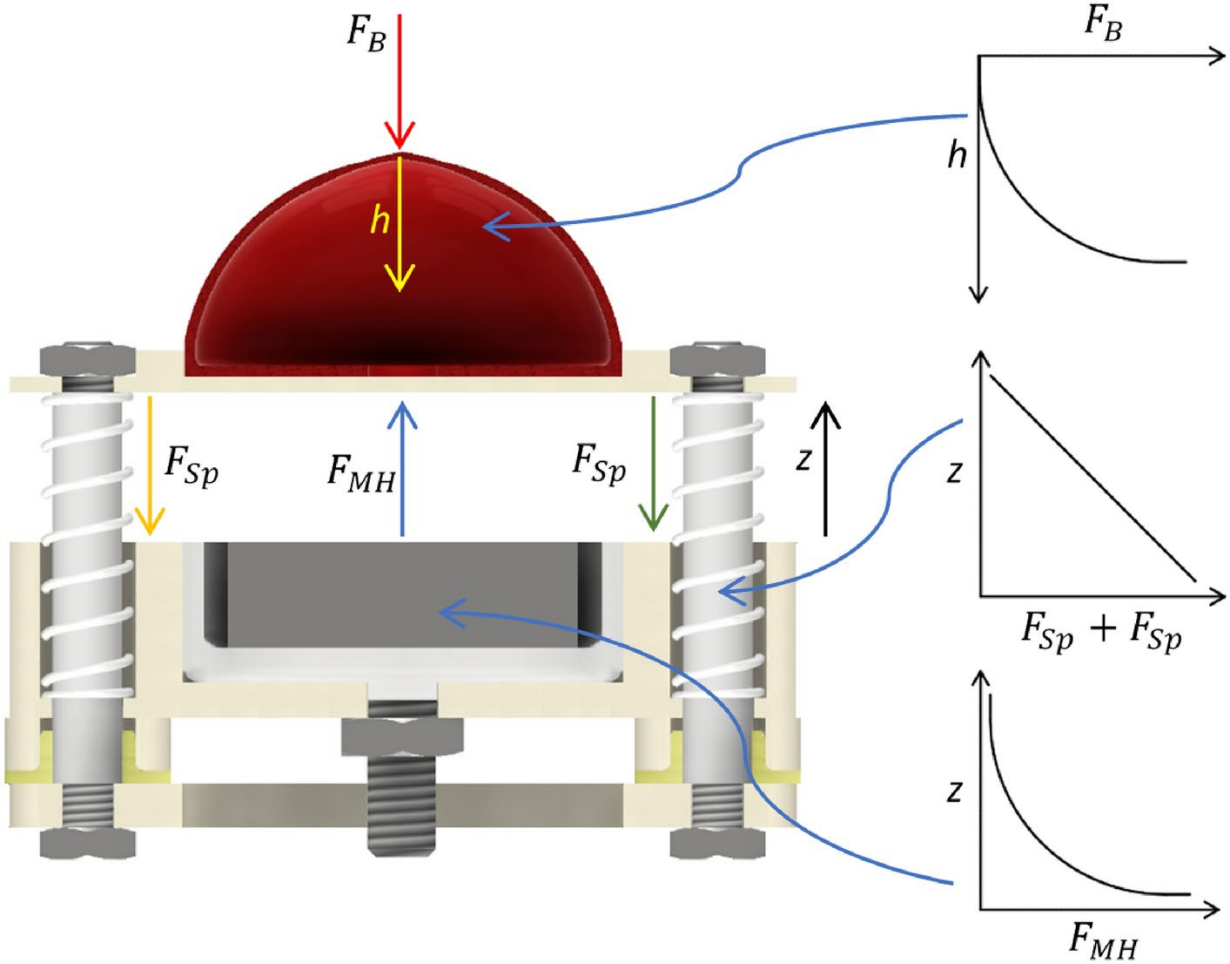
Model of a gripper jaw with indication of the deformation models of the various jaw components.

## Results

### Simulation model

Figure 2 shows the forces of attraction of the permanent magnet *F*_*MH*_ [N] and the forces of a pair of springs (*F*_*Sp*_ [N]), as a function of the distance of the cushion from the face of the magnetic field source *z* [mm]. The response characteristics of the two tested springs, working in pairs, are included. It is possible to distinguish three areas of work (relations) of the spring-permanent magnet. In the situation where the springs are in the free state (not compressed). The value of the magnetic flux densities of the magnet in the cushion area is too small to attract the cushion. As a result of the movement of the gripper jaws, the distance *z* is gradually decreases and the attraction force of the permanent magnet increases (decrease in air gap). The cushion is mounted on a plate. Its thickness and material affect the magnetic permeability, thus, the propagation of the magnetic field into the interior of the cushion is lowered. The combined thickness of the cushion and the underlying plate is approximately 1.8 mm. As the pin is plunged, the cushion moves toward the magnet, and therefore the value of the force of attraction of the cushion by the magnet increases (Figure 2). In contrast, the reduction of distance *z* affects a pair of springs, which repel the cushion from the permanent magnet. The *F*_*Sp*_ spring reaction force varies linearly as a function of the deflection of the springs. The force of attraction of the permanent magnet increases as the air gap z decreases. It has been designated by *F*_*MH*_. Its value as a function of the change in the air gap is exponential^31^. For the given configurations, it then reaches values slightly above *F*_*MH*_ = 25 N. When the cushion and permanent magnet are brought sufficiently close, the force will be greater than the reaction (repelling) forces of the springs and there will be an attraction of the permanent magnet to the cushion. When the jaws of the gripper are opened, the object slides out of the cushion, and the opposite phenomenon occurs. The cushion remains attracted to the magnet (*z* = 0 mm), although the force exerted by the jaws has been reduced. The result is the occurrence of hysteresis, which is visible in the waveforms of force changes, described later in the paper.

**Fig. 3:**
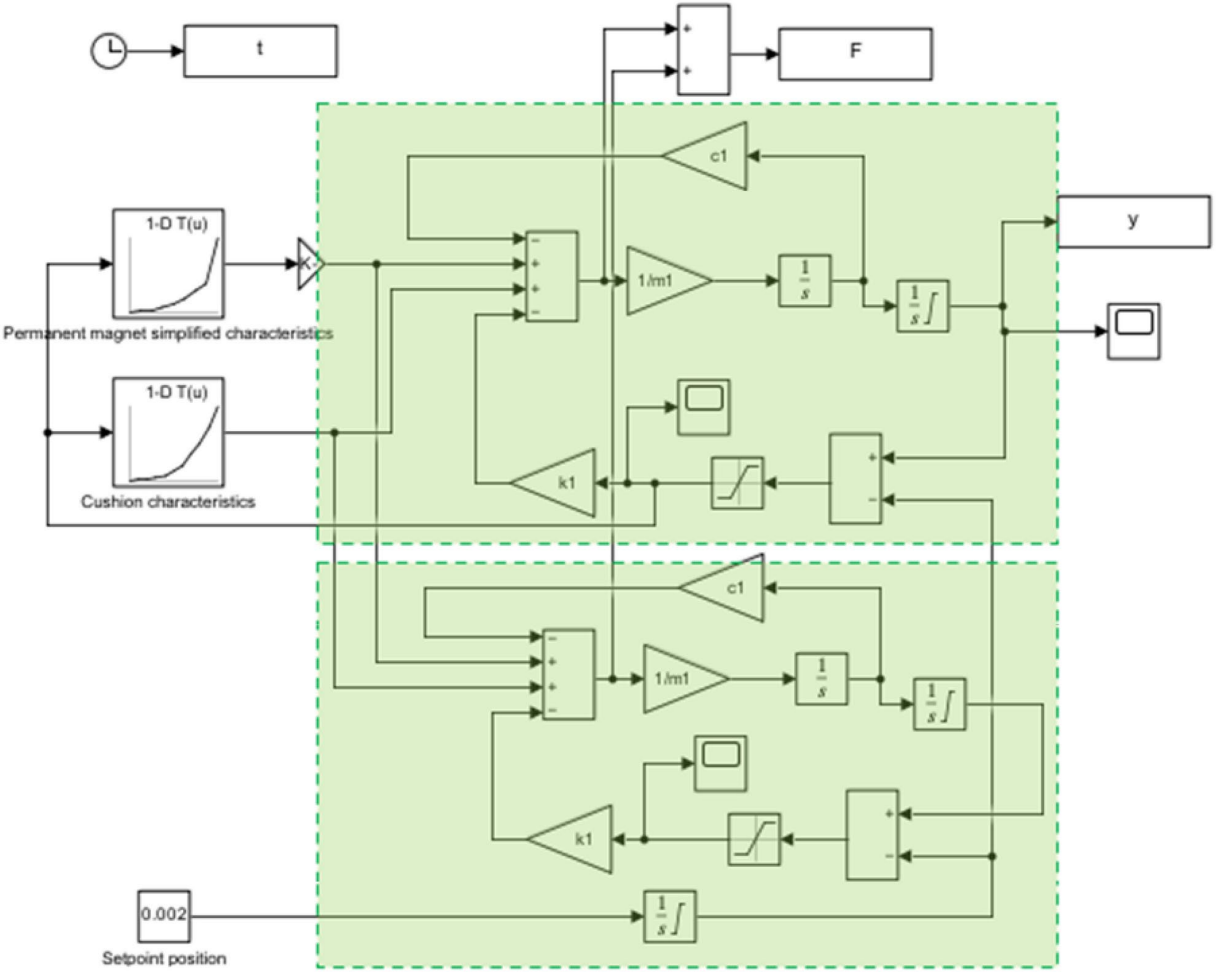
MATLAB Simulink simplified mechanical model.

As part of the research, a simplified simulation model was built in the MATLAB Simulink environment (Figure 3). The model consisted of three basic elements: a cushion model, a permanent magnet model, and a spring assembly. The cushion model and the permanent magnet model were built on a constant characteristic where the input signal is the position change and the output signal is the force value. To simplify the model and due to difficulties in identification, the permanent magnet model does not account for hysteresis. The determined force values were fed into the spring model. The basic parameters of each spring included in the model are based on catalog data.

**Fig. 4:**
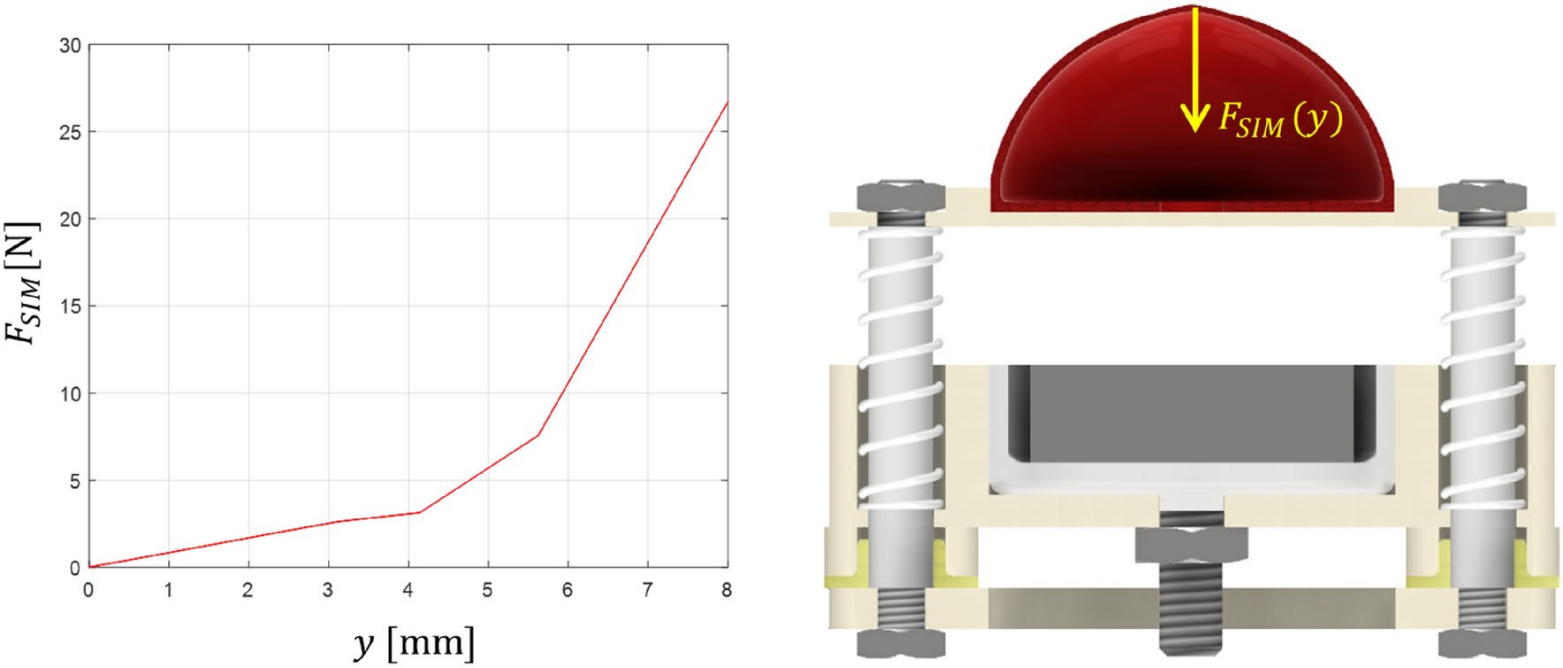
The value of force as a function of displacement determined from the simulation model.

Figure 4 shows the relationship between the change in the force value *F*_*SIM*_ [N] and the displacement *y* [mm]. There is a visible increase in the force value at the moment of compression.

**Fig. 5:**
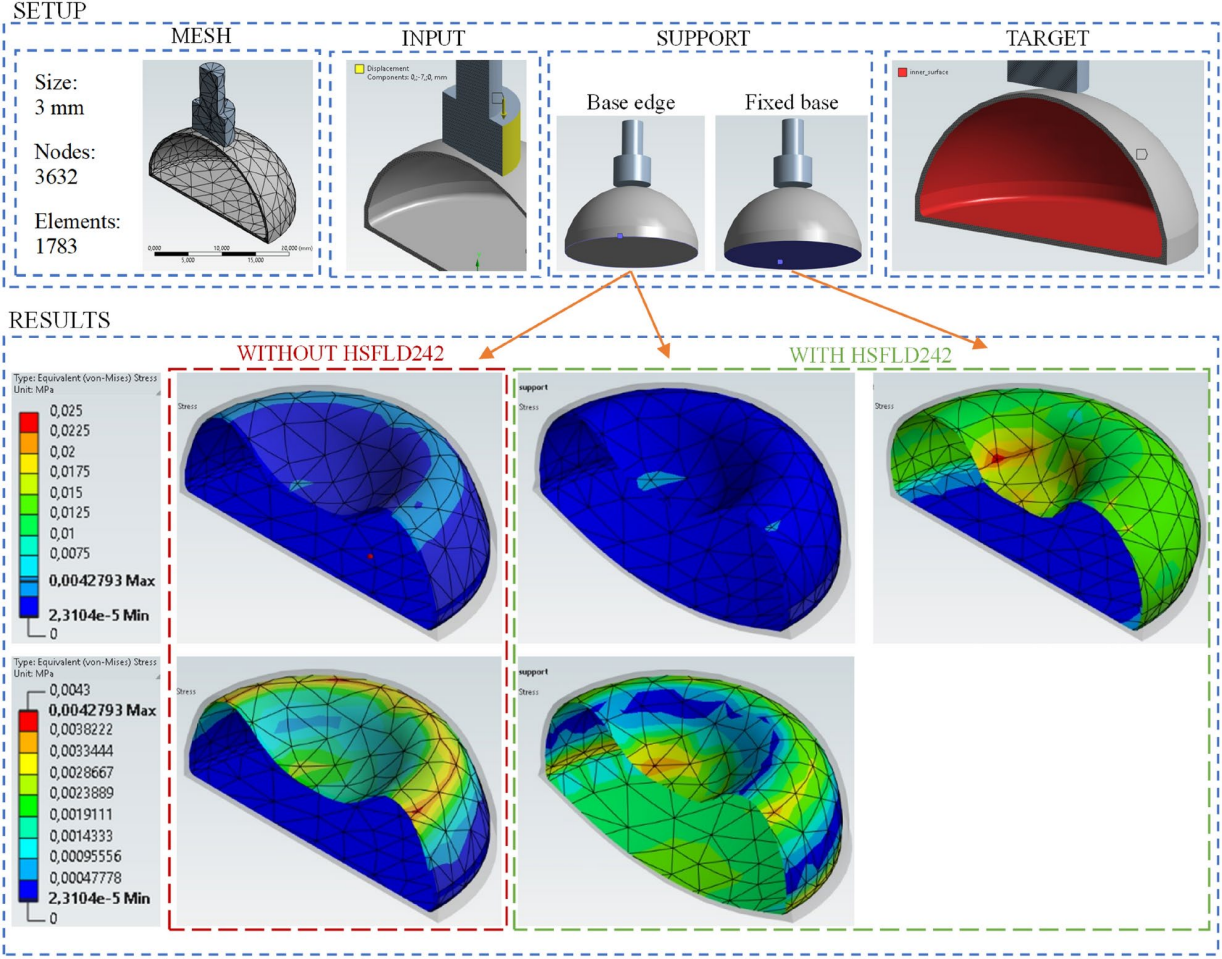
Parameters of the simulation model of deformation of the air-filled cushion and the results of the simulations carried out. Numerical model of air-filled cushion and pin with visible mesh generated in Ansys Student Workbench software.

To ensure proper performance of the gripper, in the absence of plunging force $\textit{F}_{\textit{B}}$ on the cushions by the jaws, the spring forces repelling the magnet from the cushion must be slightly smaller than the attraction force. In this way, it is possible to push the cushion away from the permanent magnet on its own while removing the external force *F*_*B*_ [N]. However, it is not recommended to introduce hard springs, whose reaction force will be much higher at maximum deflection than the attraction force of the permanent magnet. This is due to the limitation of the proposed solution, which is to obtain the appropriate force needed to plunge the object into the cushion. The higher the reaction force of the springs, the higher the plunging force must be applied to reduce the distance between the cushion and the permanent magnet.

The axial pin that plunged into the cushion was assumed. The deformation of the cushion depends on the force, originating from the indentation of the object, whose displacement causes the fluid to move. In its initial state (without indentation), the cushion has a certain volume. It is made of a material called hyperelastic^9^. As the pin plunges into the cushion, there is an increase in the inside pressure. Causes a deformation of the cushion’s walls. The mentioned hydrostatic pressure is present, acting on the inner walls. Documentation of the filaments used does not include characteristics of Shear-stress distribution. Therefore, the authors decided to follow the same appro ach as the authors of papers^9,45^, who used the 5-parameter Mooney-Rivlin model, described in the article^46^. The parameters of the FEM model of the cushion include a 3 mm, with 1783 elements and 3632 nodes (Figure 5). The embedding of the outer walls of the object in the dome of the cushion was defined as the friction of solids with a coefficient equal to 0.2. Pin displacement is an input extortion, i.e. plunging it in the center of a Y axis by 7 mm. As a result, an increase in the pressure of the fluid, located inside the cushion is present. The model also takes into account the effect of gravity in the Y axis, according to equation (1). As shown in Figure 5 two types of support have been applied, that is, base or base edge. To model the pressure inside it, HSFLD242 elements and the APDL programming language were used. The cushion was assumed to be filled with air. The change in pressure was estimated on the basis of the difference in volume of the models before and after pin plunging. The simulations resulted in a volume change of 1.7%, comparing the undeformed and deformed models.

### Experimental test - pin plunging

To verify the theoretical assumptions and simulation studies, as well as to check the correctness of the gripper operation, a test rig was built, on which a series of experimental tests of plunging the pin into the cushion filled with MR fluid were carried out. It was assumed that these tests would provide information on the value of the force required to deform the cushion. This is an important aspect with respect to gripper performance. This force should be relatively small, with as much deformation of the cushion as possible. This relation determines the ability to grasp soft or fragile objects without the need to provide a high compression force from the jaws.

**Fig. 6:**
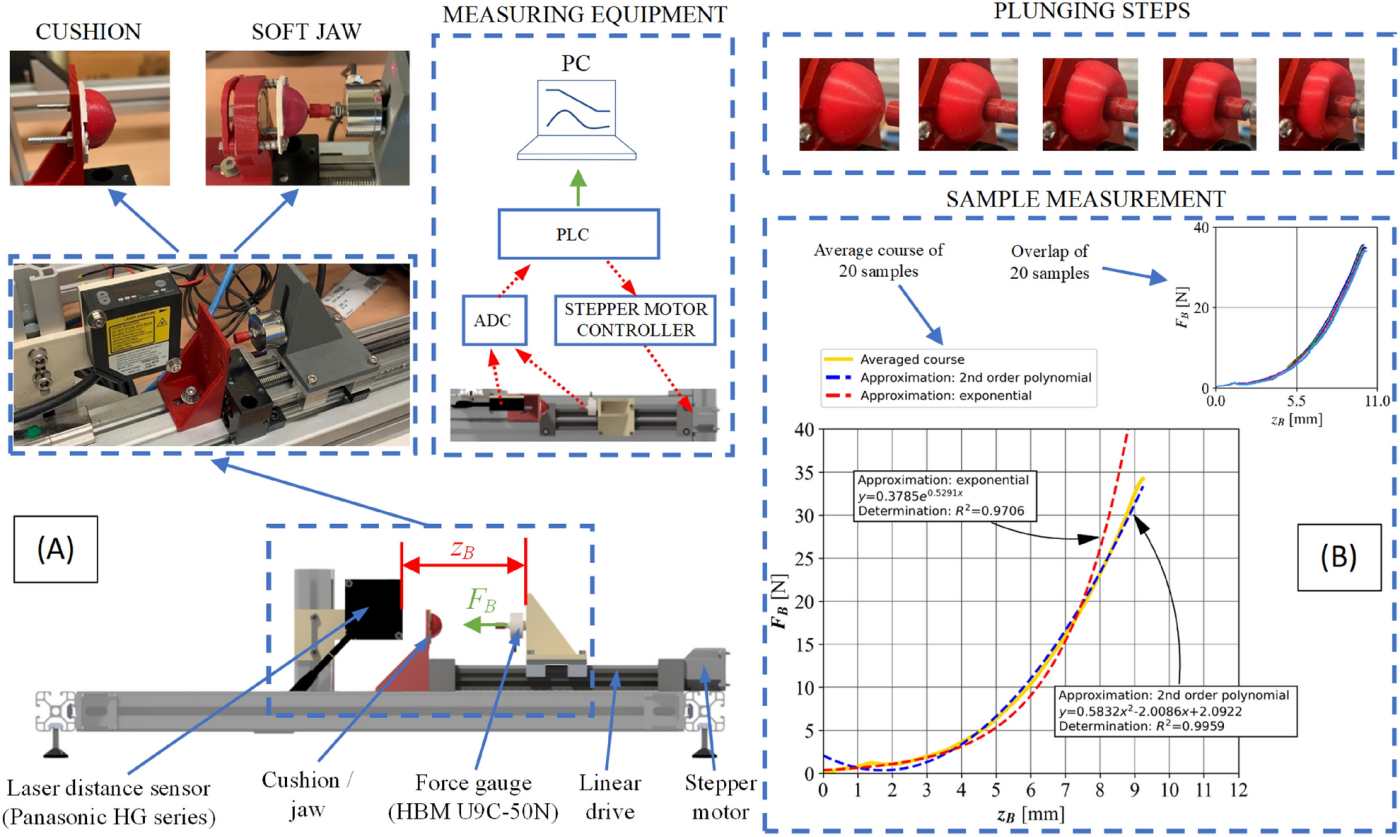
Plunging force ${F}_{B}$ measurements: (**A**) Test rig; (**B**) average course plot with approximations and 20 samples repeatability.

The primary criterion for evaluating the cushions is the obtained value of the plunging force ${F}_{B}$ for a given displacement. A schematic of the designed test rig is shown in Figure 6. The measurement system consists of a Panasonic HG series laser distance sensor and a HBM U9C-50N force sensor mounted on a linear drive with a stepper motor. The sensors are connected to a B&R PLC through an ADC input. A 16-bit measurement converter was used. The stepper motor is operated by a dedicated controller. The data was recorded on a PC connected to the PLC at a frequency of 1 kHz. Figure 6 also shows an example of a measurement performed on the test rig. During the tests, the plunging force ${F}_{B}$ [N] was recorded, depending on the pin displacement ${z}_{B}$ [mm]. The maximum value of the measured force was about 35 N. Several measurements were taken each time for the cushions. Figure 6B shows the 20 measurements obtained in the ${P}_{W4}$ cushion tests. It indicates the high repeatability of the measurement and the behavior of the cushion. Two approximations were determined: a 2-degree polynomial and an exponential function. The coefficient of determination was 0.996 and 0.971, respectively. Therefore, these can be used to build nonlinear models of the MR fluid cushion.

**Fig. 7:**
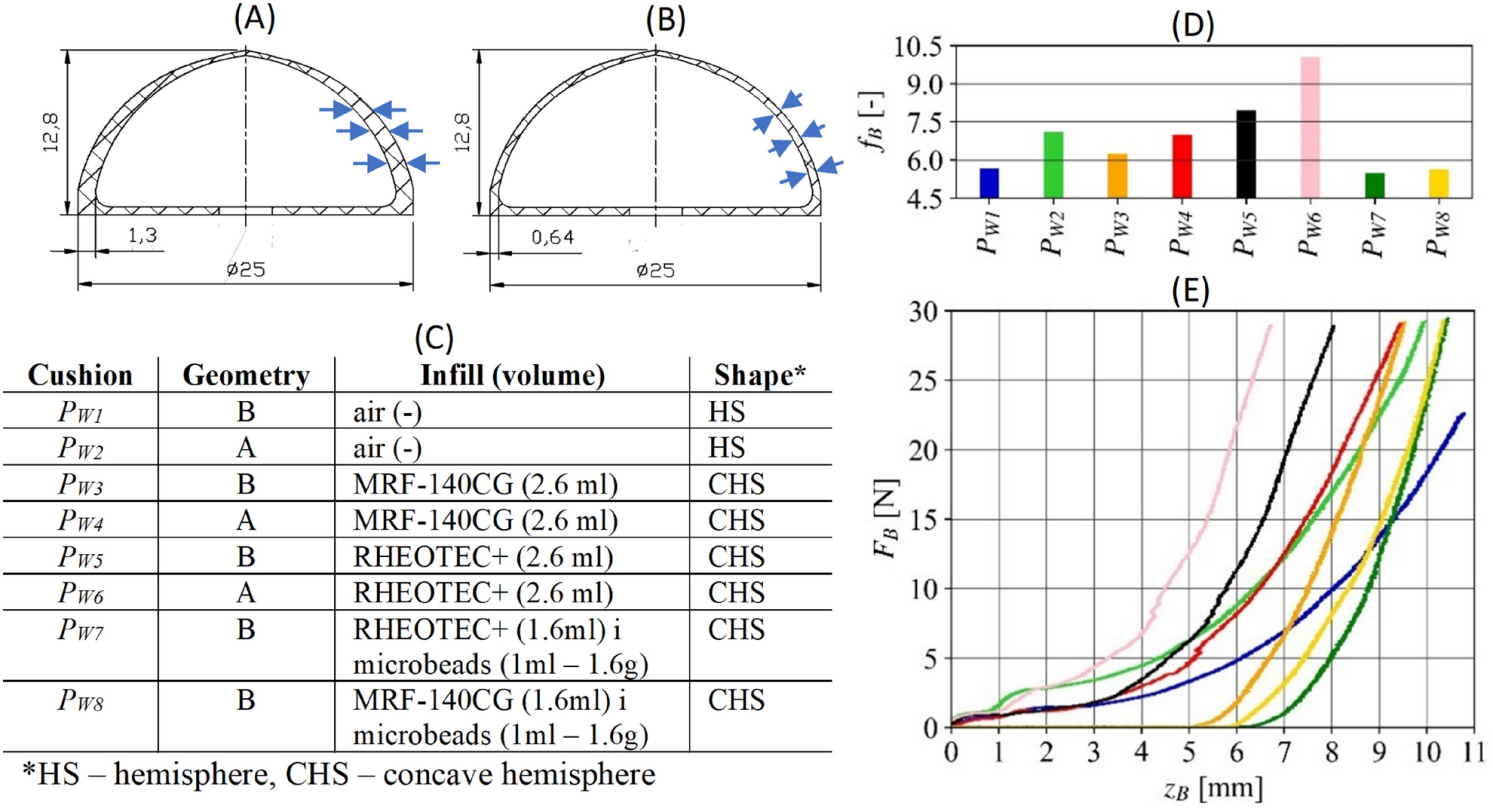
Results of the conducted tests of the forces occurring during the pin plunging of cushions with air and MR fluid: (**A**) geometrie of a 3D printed cushion with a width of three layers in cross-section; (**B**) geometrie of a 3D printed cushion with constant wall thickness; (**C**) cushion variants^47^; (**D**) adaptability coefficients of the tested cushions, (**E**) plunging forces as a function of pin displacement.

For the purpose of the study, the cushions filled with air and MR fluid were made in two geometric variants (Figure 7A, B, C). Cushions ${P}_{W1}$ and ${P}_{W2}$ were air-filled (sealed). The cushions ${P}_{W3}$, ${P}_{W4}$, ${P}_{W5}$, ${P}_{W6}$ were then filled with MR fluids (MRF-140CG and RHEOTEC+). It was decided to enter a volume of 2.6 ml of fluid, thanks to the refinement of its manufacturing and filling procedure^47^. Cushions ${P}_{W7}$ and ${P}_{W8}$ were filled with a volume of 1.6 ml of MR fluid and 1 ml (1.6 g by weight) glass microspheres, with a diameter in the range of 200-300 $\mu$m. The application potential of microspheres has been discussed in detail in articles^44,48^. Geometry in Figure 7A has a width of three layers in cross-section. Geometry in Figure 7B has a constant wall thickness. The same cushions were also used in the other gripper research within the article^47^.

The results of the pin penetration tests for each cushion are shown in Figure 7E. They were limited to 30 N and a displacement ${z}_{B}$ not exceeding 11 mm, given the geometry of the cushion. It can be seen that for the cushions with the largest concavity, the force begins to increase after 5 or 6 mm with respect to the others. This is due to the later initiation of contact between the cushion and the pin. The primary criterion for evaluating cushions, is the achieved force value for a given displacement. The concept of stiffness was introduced, which is generally defined as follows: if the displacement of the indenter is small and the force has a high value, the cushion is characterized by high stiffness. Among the cushions tested, ${P}_{W6}$ has the highest stiffness. On the other hand, a large displacement while obtaining a low contact force characterizes a cushion with low stiffness. Among the variants tested, the ${P}_{W1}$ cushion is the best, but the ${P}_{W7}$ and ${P}_{W8}$ can also be considered. For the collected set of measurements, it can be proposed to introduce a stiffness factor ${F}_{B}$ (Figure 7D). It is the sum of the four ratios of the values of the plunging force and certain displacement of the pin:

$$\begin{aligned} \textit{f}_{\textit{B}} = \mathop {\sum }\nolimits _{i=1}^{4} \frac{F_{B_{i}}}{z_{B_{i}} F_{B_{i}}} \end{aligned}$$

where: ${z}_{{B}_{i}}$ [mm] value of pin displacement for a given value of plunging force ${F}_{{B}_{i}}$ [N], whereby:

$$\begin{aligned} \textit{F}_{\textit{B}_{\textit{i}}} \in \{5,10,15,20\} \end{aligned}$$

The higher the value of the displacement of the pin, i.e. the farther the point at which a given force is obtained, the less adaptive the cushion is. Consequently, the lower the value of the proposed coefficient, the more favorable it is. Cushion configurations with the same filling, but two different geometries, are also worth noting. Thinner walls allow less work ( taking the force and displacement of the pin) to deform the cushion.

**Fig. 8:**
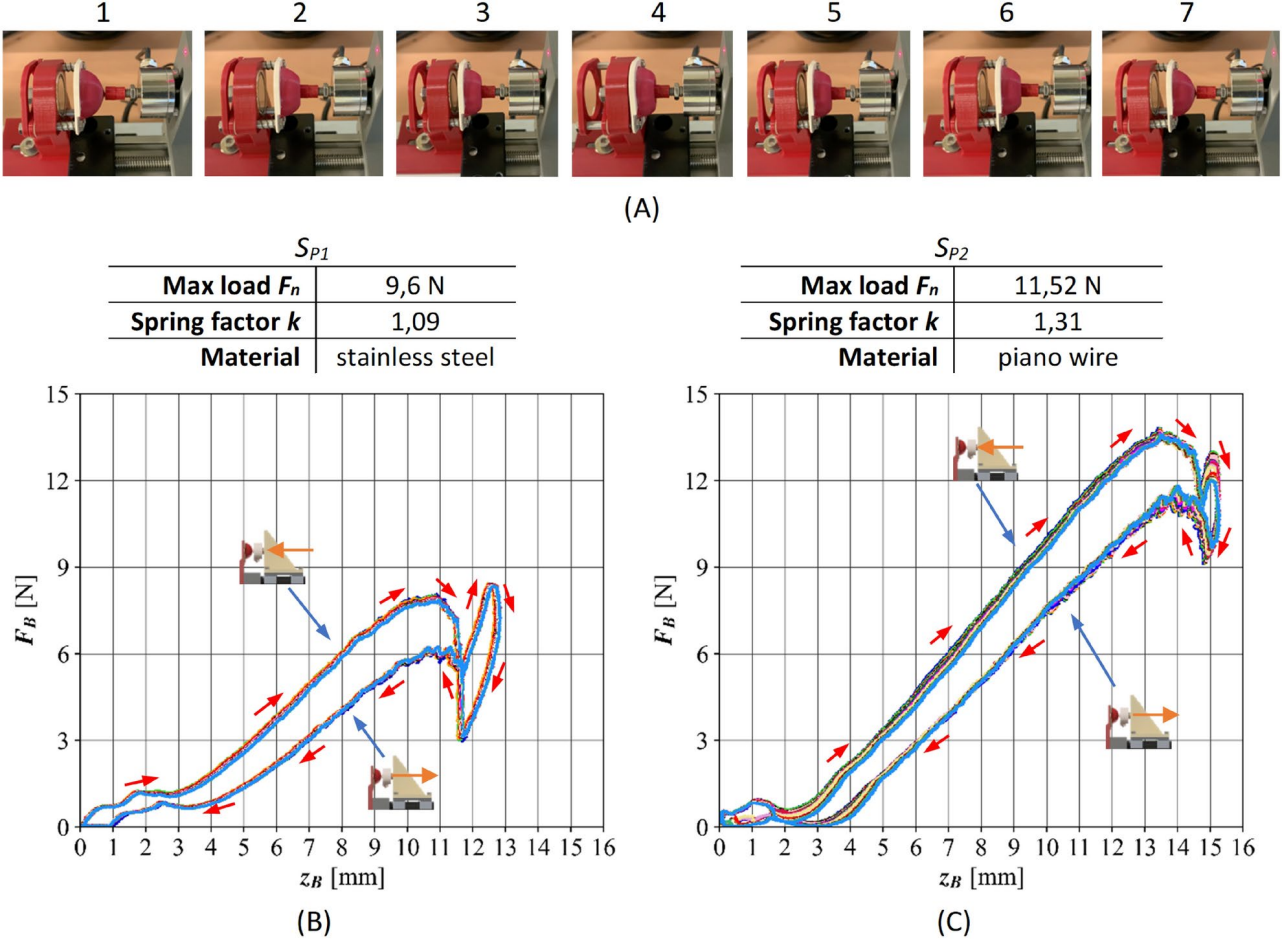
The measurements of the plunging force *F*_*B*_ for the jaws (colours indicate 20 samples): (**A**) individual movement sequences, (**B**) spring *S*_*P1*_, (**C**) spring *S*_*P2*_.

Figure 8 shows an example of the plunging force exerted by the pin on the jaw with the MR cushion. Figure 8A shows the following steps of the process of plunging and pulling out the pin for the jaw with the ${P}_{W8}$ cushion. The process begins with the initiation of pin-cushion contact, which then leads to the deformation of the cushion. In the next steps, further plunging of the pin into the cushion was observed, up to the point marked by the first vertical line in blue. Next, there is a linear deflection of the springs, which continues until the point marked in the figure with the second blue vertical line. This is followed by a reduction in the contact force, due to the permanent magnet’s attraction of the cushion containing ferromagnetic particles. In the next stage, after crossing the third vertical line in blue, the plunging of the pin occurs again. Figure 8B and 8C plot 20 measurements for each pair of springs.

Displacements for the ${S}_{P1}$ springs are shorter, due to the lower reaction force. Therefore, the cushion’s plunge phase is shortened and the deflection of the springs occurs faster. The characteristic at the corresponding point is more “flat”, which indicates simultaneous cushion depression and spring deflection. Analyzing Figure 8, it is worth noting the good repeatability of the jaw response characteristics. The plots reveal hysteresis, caused by the attraction of the cushion by the permanent magnet. The geometric parameters of the springs are as follows: outer diameter 5.33 mm, inner diameter 4.21 mm, length in free state 17.53 mm, maximum stroke 8.74 mm. The others are included in Figure 8. Dimensions of permanent magnet: 19.2 x 6.1 mm and yoke: 25 x 8 mm, width 1.9 mm.

### Experimental test - object transfer properties

**Fig. 9:**
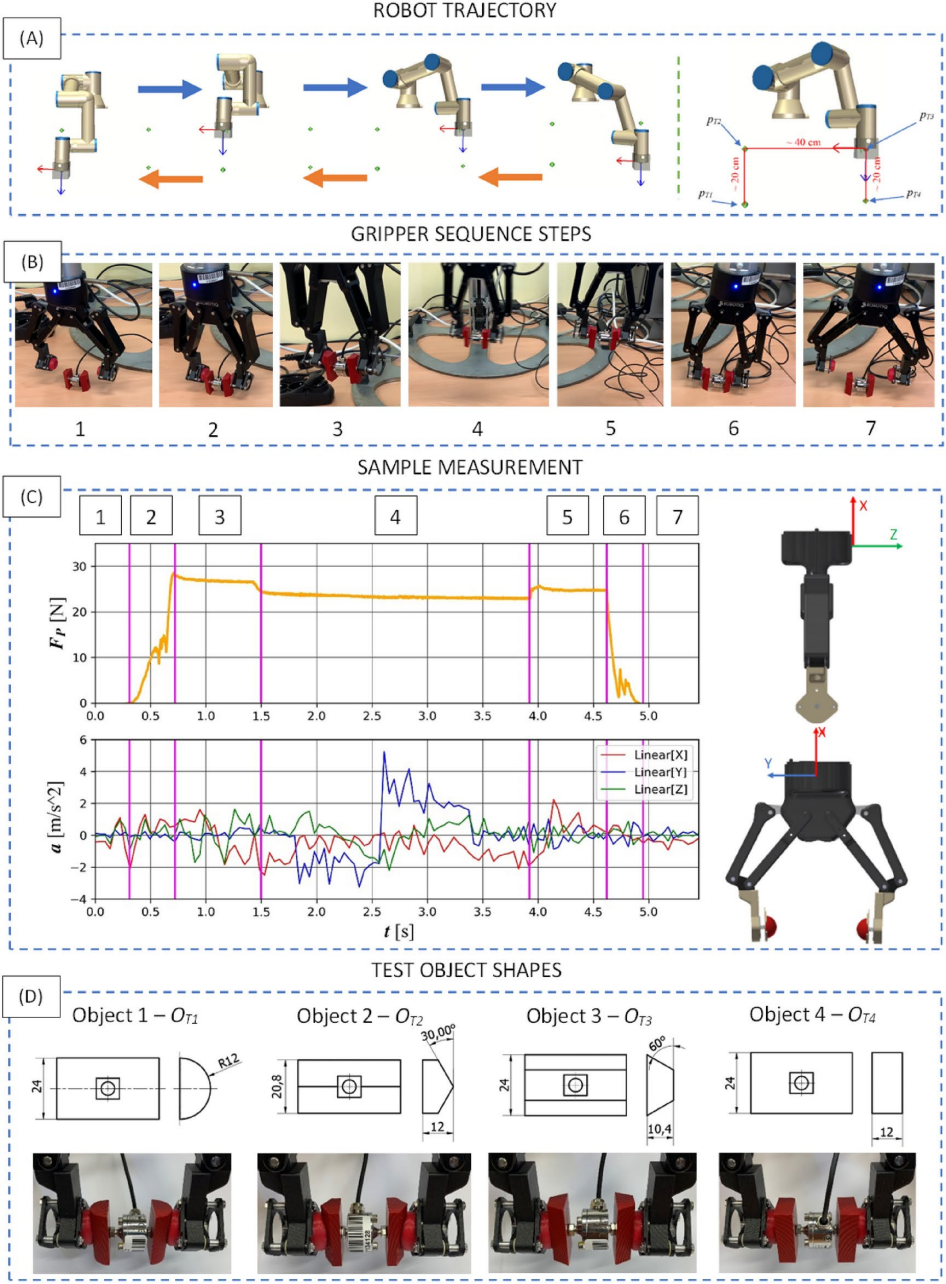
Object transfer experiments prerequisites: (**A**) the trajectory of the robot’s movement during the test procedure; (**B**) gripper sequence steps during motion, (**C**) an example of the course of the recorded value of the squeeze force during transportation, indicated phases: 1 - no contact, 2 - grasping with squeeze, 3 - lifting up an object, 4 - horizontal transportation, 5 - lowering the object, 6 - releasing the object, 7 - no contact; (**D**) objects used in the study.

The methods of testing grippers to determine their effectiveness for moving objects are based on statistical determination of the number of correct transport attempts. Usually, the only information from the results is the percentage of positive attempts to move the object^44^. With the criterion for passing, the attempt is the transfer of the object from namely point ’A’ to point ’B’. The object falling out of the gripper’s embrace results in marking the attempt as negative. The publication^44^ introduced a movement trajectory consisting of lifting an object to a height of 200 mm, transporting it horizontally for a distance of 400 mm and lowering it from a height of 200 mm. Based on data from the literature, the following measurement method was proposed. The same motion trajectory was used (Figure 9A and 9B). The robot starts its movement at a point designated as ${p}_{T1}$. It then closes the jaws of the gripper, grasping the object. The robot lifts it to the point ${p}_{T2}$, which is at a height of about 200 mm. The next stage of movement is to horizontally transport the object over a length of about 400 mm, to ${p}_{T3}$. The final stage of movement is the lowering of the gripper with the object from a height of 200 mm to ${p}_{T4}$. The object is released from the pressure of the jaws and, thus, set aside on the work surface. Then it is picked up again and the discussed procedure proceeds in reverse order. Object transfer is performed in a loop. Four geometries of the object to be grasped were proposed. The geometries and dimensions are shown in Figure 9D. A measurement of the jaw squeeze force on object walls was introduced (force gauge between object in Figure 9D). This made it possible to obtain information on the stability of the jaw squeeze, during the execution of the movement. Attention was also paid to the effect of changing the object’s orientation after it was put down and picked up again. This was possible because the measurements were made in a continuous loop. An object was picked up, transported, put down and picked up again. In this way, a minimum of 16 transport runs of a given object were generated. Measurements were made for different combinations of speed *v* [$^{\circ }$/s] and acceleration *a* [$^{\circ }$/s$^{2}$] of the robot’s movement in the range of 100, 200, 300, giving a number of combinations equal to 9. Considering 4 different objects 36 scenarios were obtained giving 576 samples in total. All transport attempts were successful, achieving 100% effectiveness. An example of the course of the recorded value of the squeeze force is shown in Figure 9C. The individual phases of movement discussed earlier were also indicated. They correspond with Figure 9B and are as follows: 1 - no contact, 2 - grasping with squeeze, 3 - lifting up an object, 4 - horizontal transportation, 5 - lowering the object, 6 - releasing the object, 7 - no contact. A particularly important aspect is 4th phase (Figure 9C), the horizontal movement of the gripper.

**Fig. 10:**
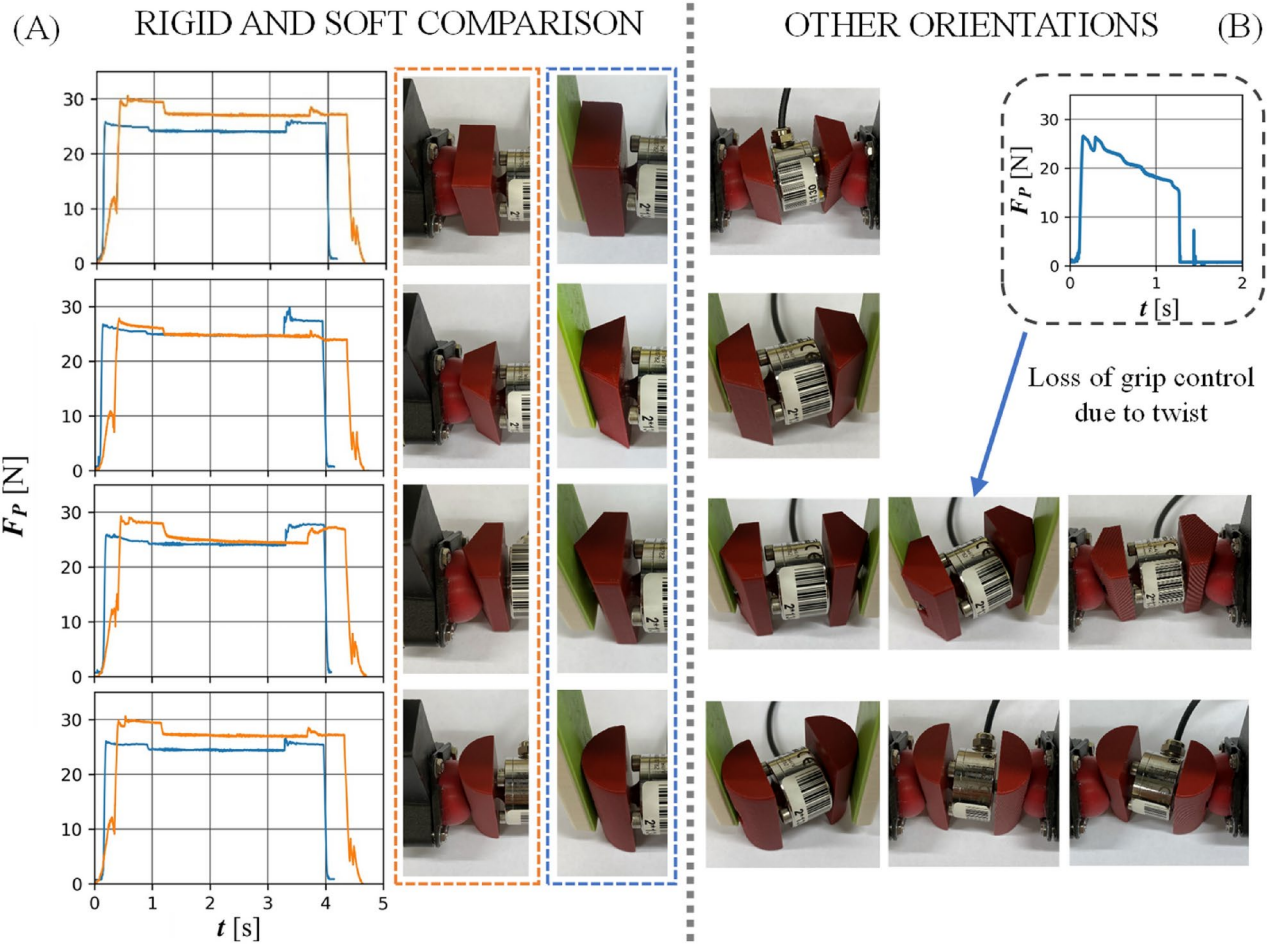
Comparison of the rigid jaw and the jaw with MR cushion for acceleration of 300 $^{\circ }$/s$^{2}$ and velocity of 300 $^{\circ }$/s.

The use of a force sensor enabled the force measurement during the process of the object handling. Based on this, methods of evaluating the repeatability and stability of the grasp were proposed. Figure 10 shows a comparison of jaw squeeze force for rigid jaws and MR cushion jaws during transportation proces for acceleration of 300 $^{\circ }$/s$^{2}$ and velocity of 300 $^{\circ }$/s. Rigid jaws allow to grasp an object sooner (maximum force is reached earlier). Consequently, their passes are shorter. Once grasped, the squeezing force of the jaws does not drop significantly and is maintained during horizontal movement. In most cases, it is minimally less than that exerted by the soft jaws. The gripping process by MR jaws is slightly extended. Soft jaws, when adjusted to the shape of the object to be grasped and transmitted horizontally, reduce the value of the force exerted. It should be noted, that Figure 10 highlights a situation in which the rigid gripper has lost grip stability due to the object rotation in its jaws.

Grip repeatability is defined as a measure of the extent to which the measurement results of the recorded force value associated with a given grip are repeatable during multiple handling of the same object, in the same orientation. It is determined by the spread of the measured readings of the force values ${F}_{P}$ of the jaws in relation to their mean value ${F}_{Pavg}$:

$$\begin{aligned} {\left. \begin{aligned} \textit{t}_{1}&= 2 s \\ n&= 16 \end{aligned}\right\} } {\textit{F}_{\textit{Pavg}} = \frac{\sum _{i=1}^{n} F_{{P}_{i}} (t_{1})}{n} } \end{aligned}$$

where, ${F}_{{P}_{{i}}}$ (*t*_1_=2s) [N] is the value of the recorded jaw force in the 2nd second of movement, *n* [-] is the number of samples equal to 16.

**Fig. 11:**
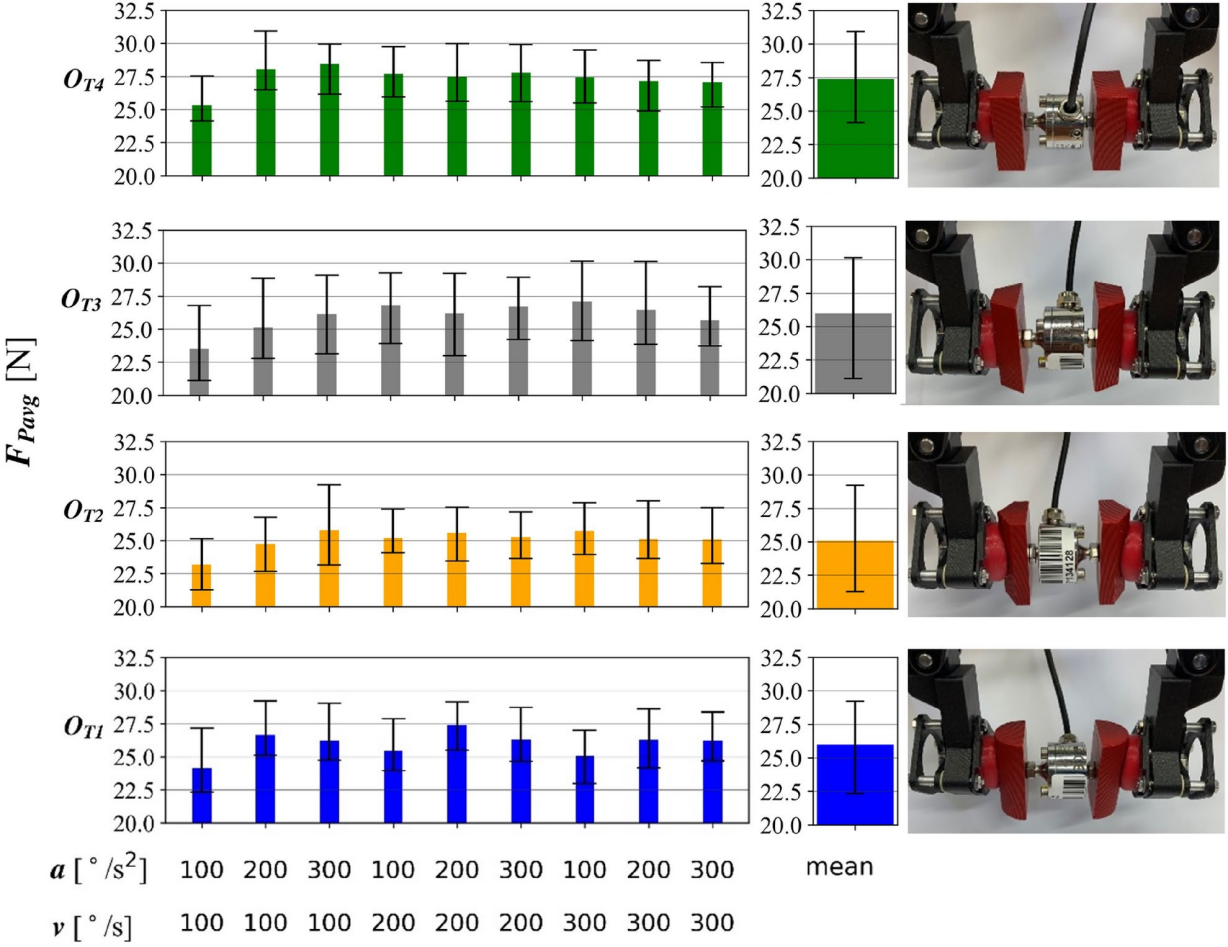
The average value of the force of the jaws on individual objects during horizontal movement, with the spread of measurement values indicated - repeatability of the grasp.

A shorter span of readings means higher grip repeatability. The results of the analysis are presented in the table in Figure 11. Force measurements for a velocity of 100 $^{\circ }$/s and an acceleration of 100 $^{\circ }$/s$^{2}$ in all object cases have the lowest mean value. The highest values of the average contact force were obtained for the ${O}_{T4}$ object and slightly less for the ${O}_{T1}$ object. The lowest, on the other hand, is for object ${O}_{T2}$. Flat side walls of the object resulted in a higher repeatability of the grip force. Sharp edges as in the ${O}_{T2}$ and ${O}_{T3}$ objects caused a slight angular deviation from the axis of the gripper pad. This was observed during the execution of the tests. The smallest values for the spread of the measurement results occur for the ${O}_{T4}$, while the highest values occur for the ${O}_{T3}$. As can be seen, different configurations of velocity and acceleration do not significantly affect the force. They should be considered as a larger test sample. However, the greater the variability obtained for each tested object, the more frequent the variation in grasping, discussed above.

**Fig. 12:**
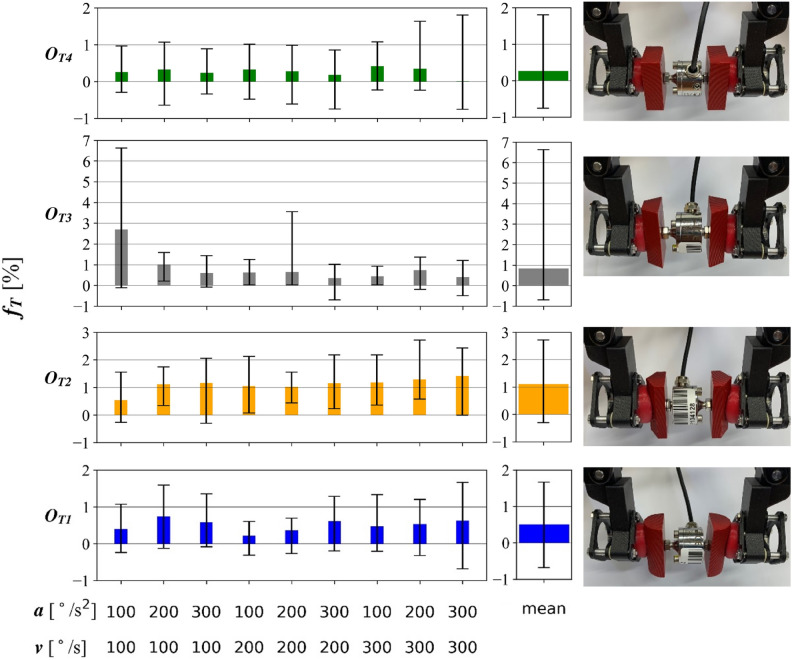
The average percentage change in the value of the pressure force ${f}_{T}$ on the object carried during horizontal movement, with the spread of the measurement values indicated, the stability of the grasp.

The stability of the grasp is defined by the average percentage change in the value of the pressure force ${f}_{T}$, occurring during horizontal movement, as an index expressed by the following formula:

$$\begin{aligned} {\left. \begin{aligned} {t}_{1}&= 2 s \\ t_{2}&= 3 s \\ n&= 16 \end{aligned}\right\} } {F}_{T} = \frac{\sum _{i=1}^{n} \frac{F_{{P}_{i}} (t_{1})-F_{P_{i}} (t_{2})}{F_{P_{i}} (t_{1})} \cdot 100\%}{n} \end{aligned}$$

where: $\textit{F}_{\textit{P}_{\textit{i}}}$ (2 s) [N] is the value of the recorded jaw force in the 2nd second of movement, and $\textit{F}_{\textit{P}_{\textit{i}}}$ (3 s) [N] is the value of the recorded jaw force at the 3rd second of movement, as shown in 10, *n* [-] is the number of samples equal to 16.

The interval between the measuring points of 1 second was experimentally selected, based on the recorded courses. The difference of the force values at these two points is divided by the force value at the 2nd second of movement. The sum of these changes is divided by the number of runs to give the arithmetic mean value. The values obtained in this way are shown in Figure 12. The MR cushion gripper has good repeatability and reliability to handle objects with defined geometries, shown in Figure 9D. Among the samples tested, the best results were obtained for handling of flat objects (${O}_{T4}$), for which the highest repeatability and consistency of jaw pressure were obtained. Slightly weaker results were obtained for the ${O}_{T1}$ object, characterized by rounded walls. The transfer process of the ${O}_{T2}$ object was characterized by a repeatability comparable to that of the ${O}_{T1}$ object, with the lowest values of the mean contact force. For the ${O}_{T3}$ object at a of 100 $^{\circ }$/s$^{2}$ and v of 100 $^{\circ }$/s, six runs were obtained in which the jaw force decreased significantly (Figure 12). However, this was not a recurring trend, so they can be considered as outliers. The coefficient values confirm the observations of the results of measuring the force of pulling objects from the jaws.

**Fig. 13:**
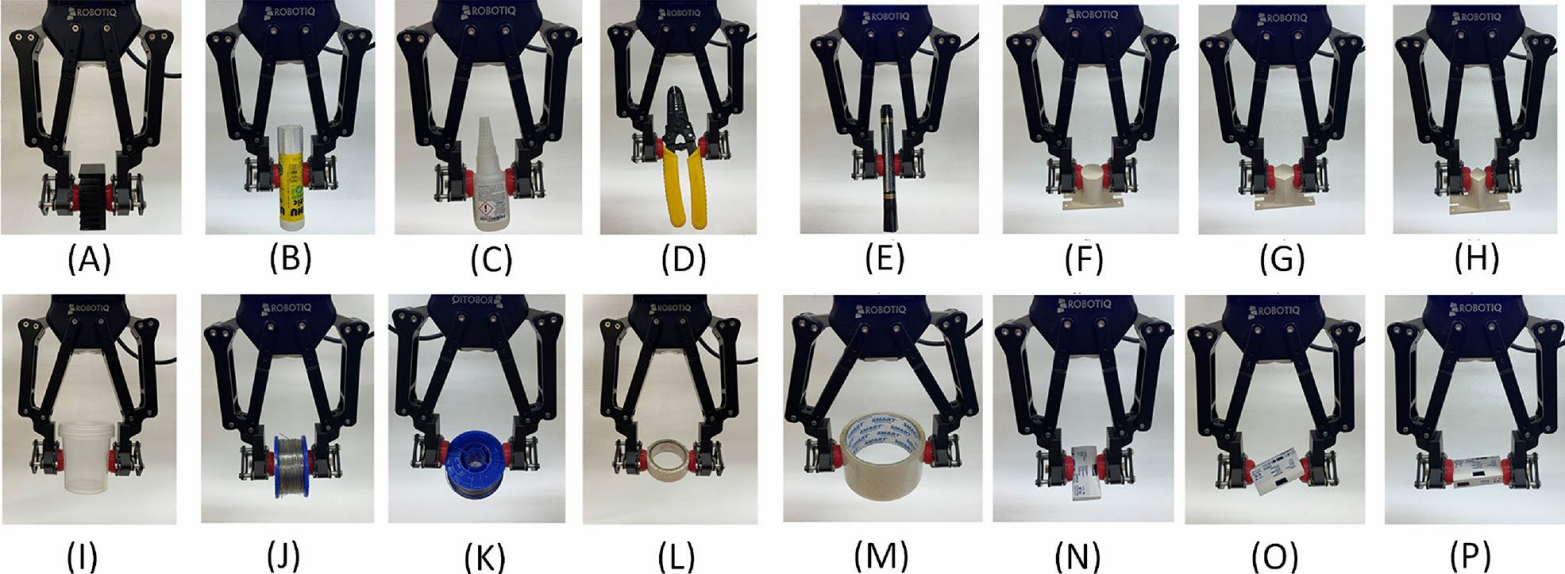
Other objects grabbed and carried in the tests performed: (**A**) aluminum heat radiator; (**B**) glue stick; (**C**) glue in a bottle; (**D**) wire stripping tool; (**E**) pen; (**F**) circular object; (**G**) hexagonal object; (**H**) square object; (**I**) sample container; (**J**) solder binder orientation no. 1; (**K**) solder binder orientation no. 2; (**L**) tape; (**M**) packing tape; (**N**) box orientation no. 1; (**O**) box orientation no. 2; (**P**) box orientation no. 3.

Furthermore, experiments were also carried out to grasp and moving other objects, which are shown in Figure 13. It was aimed at verifying the grasping of various objects in terms of shape, surface structure, or material. Most of them were handled with the MR gripper without any difficulty. An interesting object is a cuboid box, which was successfully gripped in three different positions (Figs. 13N, 13O and 13P).

## Discussion

It is crucial, for the correct operation of the developed gripper, that the shear stress in the MR fluid be high when the permanent magnet is close to the cushion and as low when it is far away. This allows the cushion to become “soft” when the object is in its area, which in turn allows its shape to adapt to the object. When the jaws are closed and the magnetic field is inserted into the cushion volume, there should be as much stiffening of the MR fluid as possible, providing a more stable grip. Two cushion geometries were proposed. The next part of the article involved experimental testing of the cushions. The cushions were tested both before and after filling with MR fluid. For this purpose, a dedicated test stand was prepared.The force required to deform the cushions and the gripper jaw was measured. This allowed them to be compared with the proposed cushion filled with MR fluid. The results made it possible to determine the effectiveness of using MR fluid in the gripper cushions. In the final stage of the work performed, the performance of the jaw gripper with MR cushions was checked on the UR3e robot. To evaluate this gripper, the method of measuring the effectiveness of object handling, presented in^44^, was used. It was extended to measure the pressure force of the jaws on the transported object. A method was proposed to evaluate the repeatability of object handling.The research showed that the proposed gripper with MR cushions obtained the best results when handling objects with flat and rounded shapes. When the contact force during the movement, it was possible to register the changes seen in the case of objects with sharp edges. Although all transfer attempts were successful, a tendency was observed to decrease the value of the pressure force of the jaws acting on an object with sharp edges, during horizontal movement.With MR fluid cushions, the maximum drag force occurring during ejection is gradually achieved gradually as the object moves in the jaws. There is a certain range of movement in the ejection direction in which the object can move quite freely (while under slight pressure from the cushion), before it moves toward the local area where there is greater stiffness of the MR fluid inside the cushion. This feature of the proposed solution is positive, as there is a certain range in which the object is in compression and will not be damaged during the transfer.

**Table 1:** Comparison of selected grippers.

Lp.	Gripper type	Nominal dimensions of the gripping element	Maximum pullout force [N]	Comment
^7^	Based on tentacles with tabs	No data	10; 21, 40	The value of the pulling force depends on the pressure applied to the tentacles. The higher its value, the more difficult it is to pull the element out of its embrace. The test object was a 3D printed sphere, no data on the dimensions of the
^14^	Cushion filled with granes	Cushion diameter of 46 mm	3,6; 23,6; 38	They showed a strong dependence of the size of the gripped object in relation to the diameter of the gripper, which further changes with different volumes of filling the cushion with coffee beans. The authors of this article also noted the need for an external force on the gripper side. They determined the results by the ratio of the object’s pulling force to the force required for application.
^26^	Based on flexible jaws	Jaw height 110 mm, width 44 mm, thickness 20 mm, wall thickness 2 and 1 mm	42	The gripper, due to its design, is able to maintain a high level of pulling force values as a function of the displacement of the object held. The only object that was subjected to the pullout test was a cylinder. No data is available on its dimensions. The scale of the images shown suggests about 80 mm in diameter.
^44^	Jaws with flexible MR fluid cushions	Yoke with a diameter of 40 mm and a height of 20 mm. The distance between the cushions is 70 mm.	17; 21; 30; 51	The value of the pullout force depends on the shape of the grasped object. Tests were performed in the absence and with the presence of a magnetic field in the cushion volume. Twice the pullout force values were obtained for an object with a spring and spherical geometry. There was a negligible difference for a cylinder and a prism. In this case, the variation in the dimensions and size of the individual objects.
^40^	MR fluid cushion	Base diameter about 108 mm, height about 38 mm	58	The value was obtained for an applied force of 40 N. The gripped object was a cylinder with a diameter of about 44.5 mm. The authors of the article also used the relation of the pullout force to the force required to be applied by the gripper as in [48].
^49^	Based on the suction cup	Suction cup diameter 20 mm	1,2; 3; 7; 7,5	The grasping efficiency was compared in the absence and with the presence of a magnetic field. The experiments also included the use of different volumes of MR liquid, which is introduced at the contact surface of the suction cup with the grasped object. The maximum object pulling force of 7.5 N was obtained with an applied force of 1.5 N.

Summarizing the results of the tests conducted, it was found that the MR cushion gripper shows high stability and repeatability in transportation of the objects with defined geometries (Figure 13). Among the samples tested, it performs best in the transportation of flat objects (${O}_{T4}$), for which it achieves the highest repeatability and stability of jaw pressure. Slightly weaker results were obtained for object 1, characterized by rounded walls. The key to this solution are the results for the other two objects. Object ${O}_{T2}$ manifests repeatability comparable to object ${O}_{T1}$, with the lowest average pressure force values obtained for it. On the other hand, it is also characterized by the lowest grip stability among the geometries considered. However, in doing so, it is worth noting that this was the expected behavior. The MR cushion, compared to the flat-jawed gripper, performs very well. As mentioned earlier, object ${O}_{T2}$ is challenging to grasp with flat jaws, which causes it to rotate. The MR cushion allows this object to be grasped without changing its orientation, which can have an impact with a given gripper application. A very important advantage of using the MR fluid cushion is its adaptability to the shape of the object being grasped, which allowed them to be picked up in almost any orientation. The MR fluid cushion “remembers” the shape of the indenter when a magnetic field is applied.

Differences in measurement methods have a very large impact on the results obtained. Methods for testing the force of pulling out objects, as described in the literature, are usually subject to the error of failing to maintain the axiality of the measurement. They describe a variety of robotic grippers, each of which exhibits specific characteristics in the context of the object pull-out force. Research presented in the article^47^, including the use of the Robotiq 2F-140 gripper and rigid flat jaws, air-filled pads, and MR fluid. All types of jaws were brought to the same reference system (gripper base) and gripped the same test objects, allowing a reliable comparison of results. The same procedure was applied within the scope of this article. It does not always apply, but it gives the same reference and makes the results more comparable.

Table 1 shows representative examples described in the literature, in which the results of tests on the force of pulling an object from a gripper are presented. Within the “ comments” column, both the conditions of the experiments performed and summary conclusions are given. The gripper jaws studied as part of this dissertation are significantly smaller than most of the solutions mentioned in the literature. It is extremely important to identify the constraints on the grasped objects that determine the capabilities of the gripper. This includes, for example, their dimensions^48^ and shape^44^. It turns out that each solution requires an individual objective approach to reveal its limitations. This makes it all the more worthwhile to additionally conduct experiments with traditional solutions such as rigid jaws, allowing comparison of the results obtained for a new design within the same frame of reference (e.g., the gripper body).

In general, the highest pullout forces of objects are obtained by jaw grippers and pillow grippers. The lowest pull-out force values are obtained in solutions based on suction cups and the adhesion principle. In the case of pneumatic grippers with tentacles, the pull-out force depends mainly on the given pressure. In turn, two articles pointed out that the pulling force strongly depends on the shape and dimensions of the gripped objects. The research presented in this paper also showed this trend. A common observation is also the need to apply an external force on the object, which comes from the gripper (mechanically clamped jaws, deformed tentacles as a result of inserted air pressure, or cushion-based gripper pressure).

The developed gripper is intended as an alternative to rigid structures that lack adaptive elements. The introduced cushions extend the functionality of the jaw gripper with soft structures capable of reflecting to some extent the geometry of the gripped object. The solution presented within the framework of this article has been subjected to a SWOT analysis (Table 2).

**Table 2:** SWOT analysis of the proposed gripper.

Strengths	Weaknesses
• no need to provide a control module, the gripper can work with the factory software;	• the gripper requires an initial force from the jaws to deflect the springs and insert the magnetic field into the cushion volume;
• low construction cost and complexity of the device - the solution can be implemented quickly;	• the need to deflect the jaw springs increases the time it takes to grasp an object (Figure 10);
• low weight of the device compared to other solutions;	• the dimensions of the cushion limit its adaptability or functionality (e.g., when gripping flat or large objects);
• ability to equip any jaw gripper with the device after prior attachment;	
• adaptability to the shape of the object to be grasped, which allowed them to be grasped in almost any orientation;	
• the MR fluid cushion “remembers” the shape of the indenter after the insertion of a magnetic field;	

Opportunities	Threats
• development of a built-in version to prevent locking of the mechanism;	• danger from damage to the cushion during operation of the device, which may lead to contamination or harm to the working environment;
• conducting further design work on stiffening the structure;	
• use of other materials for the components of the gripper structure;	

## Conclusion

Within the scope of this paper, a series of simulation and experimental studies have been conducted on the potential of using MR fluid, in a flexible cushion, as a jaw gripper. The article presents the design of a jaw using a spring-permanent magnet mechanism with a cushion made of TPU and filled with MR fluid and air (Figure 1). A jaw simulation model (Figure 2) and the deformation of the cushion (Figure 3) were presented. Two cushion geometries (Fig. 5A and 5B) and several configurations (Fig. 5C) were indicated. A test stand (Fig. 4) was presented and experimental tests of the cushion and jaws were performed (Figs. 5E and 6). The repeatability of the measurement on the designed stand was also checked (Fig. 4). As part of the work, tests were carried out on the handling of various objects (Figures 5D and 11), along a specified trajectory (Figures 7A and 7B), during which the force of the jaws on the transported object was recorded (Figure 7C). Repeatability of the grasp (Figure 9) and the stability of the grasp as an indicator of the average change in jaw force during horizontal movement (Figure 10) were determined. The MR cushion jaws were designed and prepared for use in the Robotiq 2F-140 gripper, and can also be used in any jaw gripper with a sufficiently large jaw opening range (in case of maintenance). The weight of the original Robotiq 2F-140 gripper jaw is 32.17 g, while the proposed in Figure 2 is 58.54 g. This is especially important for robots with low lifting capacity, such as UR3e. The tests have shown that the use of MR fluid cushions proposed in this work has great application potential and, after modifications, can suit industrial conditions. Methods for measuring soft structures used in grippers were also proposed. The results can find potential application in such fields as bioengineering, robotics, and precision engineering.

## Data Availability

The datasets generated and/or analysed during the current study are available in the RepOD repository: https://doi.org/10.18150/B2KCKC. https://doi.org/10.18150/GIKMCM. https://doi.org/10.18150/2ZXUYC.
